# Compassion, Discrimination, and Prosocial Behaviors: Young Diasporic Chinese During the COVID-19 Pandemic

**DOI:** 10.3389/fpsyg.2022.814869

**Published:** 2022-02-17

**Authors:** Youli Chen, Zicong Wang, Qi Zhang, Weizhen Dong, Jia Huei Chen Xu, Sizhe Ji Wu, Xiangyang Zhang, Chun Chen

**Affiliations:** ^1^Wuhan Union Hospital, Tongji Medical College, Huazhong University of Science and Technology, Wuhan, China; ^2^School of Community and Environmental Health, College of Health Sciences, Old Dominion University, Norfolk, VA, United States; ^3^Department of Sociology and Legal Studies, University of Waterloo, Waterloo, ON, Canada; ^4^School of International Studies, Wenzhou Medical University, Wenzhou, China; ^5^International School of Capital Medical University, Capital Medical University, Beijing, China; ^6^First Affiliated Hospital of Wenzhou Medical University, Wenzhou, China; ^7^School of Public Health and Management, Wenzhou Medical University, Wenzhou, China

**Keywords:** prosocial behavior, youth, COVID-19, compassion, discrimination

## Abstract

The coronavirus disease 2019 (COVID-19) pandemic has fueled anti-Asian, especially anti-Chinese sentiments worldwide, which may negatively impact diasporic Chinese youths’ adjustment and prosocial development. This study examined the association between compassion, discrimination and prosocial behaviors in diasporic Chinese youths during the COVID-19 pandemic. 360 participants participated and completed the multi-country, cross-sectional, web-based survey between April 22 and May 9, 2020, the escalating stage of the pandemic. This study found compassion as prosocial behaviors’ proximal predictor, while discrimination independently predicted participation in volunteering, and could potentially enhance the association between compassion and charitable giving. These findings suggest that prosociality among young people is sensitive to social context, and that racial discrimination should be considered in future prosocial studies involving young members of ethnic and racial minorities.

## Introduction

The COVID-19 global pandemic triggered various social issues in different countries. Although the diasporic Chinese – ethnic Chinese who reside outside of mainland China, Hong Kong, Taiwan, and Macau, constitutes a significant part of the world’s immigrant and migrant population, approximately 69 million (Zhuang, 2010; Liu and Dongen, 2013), it is a target for marginalization, stereotyping, and discrimination. Fuelled by the COVID-19 pandemic which first cases were identified in Wuhan, China, discrimination and prejudice against Asians, and particularly Chinese, have become overt worldwide. During the COVID-19 pandemic, surging anti-Asian discrimination and assaults have become a critical part of social context that diasporic Chinese need to adjust to, which may be particularly difficult for young adults and teenagers, and could challenge their prosocial development (Sanders-Phillips et al., 2009). However, we have observed widespread philanthropic disaster relief in response to COVID-19 in this population, with their main role being mobilizing and gathering donations, both monetary and material, to be used in the fight against the pandemic (ASIA PACIFIC CENTER, 2021). Chinese youths demonstrated prominent prosocialty in organizing and participating COVID-19 relief activities, such as mobilizing resources to make contributions, engaging in local Chinese communities’ donation drives, and answering to volunteer recruitments (Brown, 2020; Chan-Smitham and Tablante, 2020; Chen, 2020; Peyton, 2020).

Prosocial behaviors are behaviors intended to benefit others, which is an important aspect of youths’ prosocial development (Brittian et al., 2013). Previous studies summarized that in adolescents, prosociality is susceptible to changes as a result of cognitive and affective development, such as changes in interpersonal relations, and social context (El Mallah, 2020), which are all subject to rapid changes due to the pandemic. This being said, the COVID-19 pandemic might present as an opportunity for empowering youths and cultivating their prosociality. Recently, scholars have pointed out that racial and ethnic minority groups are underrepresented in research on prosocial development, and predictors of their prosocial behaviors warrants investigation (Armstrong-Carter and Telzer, 2021).

Using data collected from adolescents and young adults of Chinese descent during the escalating stage of the pandemic in countries including the United States (US), Spain, the United Kingdom, and etc., this study aims to advance the literature by examining the relationship among compassion, discrimination, and prosocial behaviors in this population.

### Discrimination Against Diasporic Chinese

Anti-Asian discrimination fueled by the COVID-19 pandemic hit diasporic Chinese communities disproportionately hard, posing a great challenge for youths’ adjustment.

Discriminatory incidents against Chinese have spiked in many countries other than China, taken the form of online hate speech, derogatory headlines, racial slurs, and even physical assaults and attacks (Manavis, 2020; Tavernise and Oppel, 2020), which perpetuated media, schools, public venues — almost every social aspect of youths’ lives. Correspondingly, self-reported discrimination has increased among diasporic Chinese youths compared with pre-pandemic periods (Jeung et al., 2020; Wu et al., 2020; Haft and Zhou, 2021). It is unarguable that discrimination has become a central feature of diasporic Chinese youths’ experiences around the world during the COVID-19 pandemic.

Abundant empirical evidence indicates implications of discrimination on their mental and physical health (Ahrens, 2020), socioemotional development (Grossman and Liang, 2008; Benner and Mistry, 2020) and adjustment (Juang et al., 2018). A commonly cited explanation in health-related literature of how racial discrimination impacts health is the biopsychosocial model, in which racial discrimination as a stressor elicits psychological stress responses and coping responses, and eventually leading to health outcomes (Clark et al., 1999). From developmental science literature, integrative developmental model by García Coll argues that social position variables (e.g., race, ethnicity) and its derivatives, namely racism, prejudice, and discrimination, are indispensable in understanding normal developmental processes of children of ethnic-racial groups (García et al., 1996). From a life-course theory perspective, racial or ethnic disparities in individuals’ physical, psychological, and behavioral functioning are thought to take root in adolescence, with reverberating repercussions across the life course (Sanders-Phillips et al., 2009). As such, adolescence and young adulthood present as a critical time to investigate the implications of racial discrimination.

### Diasporic Chinese Youths in Combating COVID-19

Chinese communities have been active in fighting against COVID-19 in their respective countries, and young diasporic Chinese are an indispensable part of these prosocial activities.

Young individuals and youth organizations have swiftly stepped in to disseminate information and help contain the spread of the virus. An OECD report points out that “youths are catalysts of inclusive and resilient societies” during the time of the COVID-19 pandemic (OECD, 2020). In the United States, Canada, the United Kingdom, and many more, there have been reports that Chinese youths are actively engaging in community-based prosocial activities that could help contain the pandemic, such as participating and organizing charitable donations, and collecting personal protective equipment (PPE) for local residents, hospitals, and charities. Youths have also continued to provide support in innovative (and often digital) ways. They actively run information campaigns to keep people informed on the measures to protect oneself and others, offer peer-supported mental health counseling, and share online access to educational and occupational opportunities. Youths, especially Asian-Pacific oriented ones, have shown great enthusiasm for youth-led initiatives aiming pandemic relief, such as a UNESCO-supported initiative that trained young people to investigate the impacts of COVID-19 on others of their generation, and to create policy recommendations based on their findings (UNESCO, 2020; Guay and Young, 2021). Youth Service America created a special landing page on how youths could be “contributors not spectators to the pandemic,” and provided with practical advice and guidelines (Culbertson, 2020). These charitable giving and volunteer work have been crucial to supporting vulnerable populations, addressing loneliness and anxiety, combating stigma and discrimination, and promoting social cohesion.

### Compassion and Prosocial Behaviors

In this study, we investigated compassion according to the conceptualization of Goetz et al. (2010), which defines compassion as the feeling that arises in witnessing another’s suffering and that motivates a subsequent desire to help. Specifically, compassion as an affective state consists of a brief and context-related emotional display of concern for others well-being, triggered by a clear cause, and differs from treatments of compassion as an attitude (“compassionate love”) (Sprecher and Fehr, 2005), or as a general benevolent response to others regardless of suffering or blame (“altruistic love”) (Post, 2002), or the vicarious experience of another’s emotions (“empathy”) (Smith and Lazarus, 1990). Compassion is a common emotion after crises, such as a pandemic (Zaki, 2020). Compassion is a well-established emotional antecedent of helping throughout adolescence and emerging adulthood with few contradictory findings (Lai et al., 2015), and compassion training has been found to increase prosocial behaviors in young adults (Leiberg et al., 2011).

### Perceived Discrimination and Prosocial Behaviors

Discrimination is unfair treatment that often results from stigma, or prejudice, which is a negative or hostile attitude that one has based mostly on false or incomplete information about a perceived group (Aronson and Aronson, 2018). As migrant and immigrant adolescents and young adults are gaining more social independency and exposed to more social contexts, they are also more likely to encounter racial discrimination. There are theoretical and empirical evidence suggesting that perceived discrimination may have a complex influence on prosocial behaviors, and the effects could be more pronounced in young populations with heightened sensitivity to social contexts. Previous studies have found perceived discrimination may be associated negatively with prosociality by limiting opportunities, reducing motivations, and draining cognitive and emotional resources necessary for engagement in prosocial behaviors (Twenge et al., 2007). However, further research suggested that when people feel subjected to unfair treatment based on their group membership (e.g., race, ethnicity, and gender), they incline to help in ways that make the group look more favorable in society, such as engaging in social justice movements (Smart Richman and Leary, 2009; O’Leary and Romero, 2011).

Although existing studies focused mainly on perceived discrimination’s effect on prosocial behaviors as an independent variable, we also investigated the potential moderating role of perceived discrimination as a social factor in translation from compassion to prosocial behaviors. Whilst it is often assumed that compassion will motivate prosocial behavior, Eisenberg points out that association between the two constructs are often modest and sometimes weak, and a possible reason for these modest associations is the influence of moderating variables (Eisenberg, 2000). The model outlined by Stürmer and colleages suggested one of such moderators could be group membership (Stürmer et al., 2006), and racial stereotyping and discrimination are pronounced elicitors for in/out-group membership (French et al., 2013). Goetz et al. (2010) pointed out compassion and corresponding helping behavior arise after appraisal, which is sensitive to benefits (self and goal relevance, others deservingness) and costs, which also relates closely to sense of group membership. Moreover, discriminated individuals allocate excessive cognitive resources to emotional regulation (Steele and Aronson, 1995), which consumes necessary cognitive resources for helping, discussed in the model proposed by Eisenberg and Fabes (Lockwood et al., 2014).

### Current Research

The hypotheses of this study are the following: first, perceived discrimination and compassion will independently and positively predict participation in volunteering and charitable giving in this population; second, the association between compassion and prosocial behaviors will be enhanced by perceived discrimination.

## Method

The multi-country, cross-sectional, web-based survey was conducted between April 22 and May 9, 2020. The ethics committee of Wenzhou Medical University approved the study (No. 2020-073).

### Participants

The study sample was comprised of two parts, those from snowball sampling and those from random sampling. The multi-country survey intended to include samples from countries that were major pandemic outbreak sites (e.g., the United States, Spain, etc.) and others (e.g., Japan, the United Kingdom, Australia, etc.). First, adolescents (13–17 years old) and young adults (18–25 years old) of Chinese descent who resided in a country outside of China during the survey period were recruited by snowball sampling *via* WeChat, a popular social media platform among Chinese globally. We generated the questionnaire on an online crowdsourcing platform powered by www.wenjuan.com, and enclosed a link to the online questionnaire with each invitation for participation in this survey. Then, a random sampling was done using mailing list of dingwei.netease.com, a survey company. In total, 360 valid responses were included in the subsequent analyses.

### Ethical Procedures

This research presented no more than minimal risk of harm to subjects, and informed consents/assents were collected from all adolescent (13–17 years of age) and adult (18–25 years of age) participants. All information regarding the research with contact information of researchers (C.Y., C.C.), and the voluntary and anonymous nature of participation were given to the research participants before they begin the survey. Potential participants must select a box indicating that he/she has read and comprehend the consent/assent information, and agrees to participate before they are redirected to the research survey questionnaire. The following procedures were be used to protect confidentiality and anonymity of downloaded data: (1) If IP addresses are collected by the survey tool, the addresses are deleted from the downloaded data file, and all responses were then be deleted from the online survey. The resulting data file that is used for data analysis were free of any identifiers, including IP addresses or other electronic identifiers; (2) The data files were stored on a password protected computer, and back up data files were also be stored in a secure location (C.Y.).

### Questionnaire

#### Prosocial Behaviors

Participants were presented with a multiple-choice question regarding prosocial behaviors to help fight COVID-19 pandemic (“participate in volunteer activity/activities,” and “donated money or supplies”), and were asked to endorse any helpful behaviors they engaged in. Participants could select one or more boxes; If there were no such behaviors, participants selected “none of the above.”

#### Measures

##### Compassion

Two items in Compassion subscale of Dispositional Positive Emotions Scale (DPES) were extracted and reworded to measure participants’ compassion for others in the pandemic (“I notice people who need help more often during this pandemic”; “When I see someone in need during this pandemic, I feel a strong desire to provide help”) (Cronbach α = 0.682). Respondents self-reported on a 5-point Likert scale of 0 (Strongly disagree) to 4 (Strongly agree), and a higher sum score indicates higher level of compassion.

##### Perceived Discrimination

To assess perceived discrimination, we used the Everyday Discrimination Scale (EDS) (Short Version) (Sternthal et al., 2011), which has been widely used and validated with racial/ethnic minorities in international contexts (Cronbach’s α = 0.77), and modified it to apply to the COVID-19 pandemic context. We asked the respondents how often they experienced any of the following five discriminatory situations in their daily life: (1) treated with less courtesy/respect, (2) received poorer service, (3) people act as if they wanted to avoid you, (4) called names or insulted, and (5) threatened or harassed. In the case of each item, respondents were asked to self-report on a scale ranging from 0 (Never) to 4 (Always). Sum score on the five items were used to measure levels of perceived discrimination. In this study, the 5-item scale had a Cronbach α of 0.750.

#### Sociodemographic Variables

Because the impact of sociodemographic characteristics on prosocial behaviors has been well recognized in previous work, we collected sociodemographic characteristics that have been identified as relevant variables by previous studies, and controlled for these confounders in subsequent analyses: gender, age (NCVO, 2019), place (country) of residence (GHK, 2010), educational level (Sundeen et al., 2007), employment status, marital status (Gil-Lacruz et al., 2017), immigration status (Logan et al., 2012; Greenspan et al., 2018), and language and social preferences (Sundeen et al., 2007). Additionally, we used the Oxford COVID-19 Government Response Tracker (OxCGRT) (Hale et al., 2021) as the indicator for stringency of stay-at-home orders and social gathering restrictions, which directly affect respondents’ participation in prosocial activities.

### Statistical Analysis

All the statistical analyses were performed using the Statistical Package for the Social Sciences (SPSS) 22.0 for Windows. The significance level was set at *p* < 0.05 (two-tailed).

First, descriptive statistics were generated for all variables. Chi-square test was used to detect difference in participation in prosocial behaviors among participants of different sociodemographic groups. Student *t*-test was used to compare the scores of policy stringency between those who participated in prosocial activities and those who did not.

Next, to investigate associations between prosocial behaviors and (1) compassion, (2) perceived discrimination, a series of multivariable logistic regression analyses and moderation analyses were run to examine the main effects and interaction of compassion and perceived discrimination on prosocial behaviors. The moderation analyses were constructed using PROCESS macro version 3.5.

## Results

Table 1 shows detailed information on the respondents’ sociodemographic characteristics. Overall, the study included data from 360 individuals of young Chinese descent living in 23 countries, including the United States (104, 28.9%), Spain (104, 28.9%), the United Kingdom (39, 10.8%), Japan (32, 8.9%), and other countries (81, 22.5%). Data included 177 (49.2%) males, and 183 (50.8%) females, among which the majority are students (248, 68.9%).

**TABLE 1:** Descriptive statistics of participants’ characteristics, prosocial behaviors, perceived discrimination, and compassion.

Demographics	N	Prosocial behaviors			
		Volunteering	*P*-values	Charitable donations	*P*-values
Gender			0.251		0.264
Male	177 (49.2%)	32 (18.1%)		71 (40.1%)	
Female	183 (50.8%)	25 (13.7%)		63 (34.4%)	
Age			0.268		0.561
13–17	36 (10.0%)	8 (22.2%)		15 (41.7%)	
18–25	324 (90.0%)	49 (15.1%)		119 (36.7%)	
Country of residence			0.003		0.013
The United States	104 (28.9%)	22 (21.2%)		41 (39.4%)	
Spain	104 (28.9%)	6 (5.8%)		30 (28.8%)	
The United Kingdom	39 (10.8%)	4 (10.3%)		11 (28.2%)	
Japan	32 (8.9%)	5 (15.6%)		10 (31.3%)	
Others^(a)^	81 (22.5%)	20 (24.7%)		42 (51.9%)	
Stringency	76.29 ± 13.52	71.76 ± 14.01	0.006	76.38 ± 13.59	0.927
Educational level			0.779		0.284
Less than high school	13 (3.6%)	1 (7.7%)		2 (15.4%)	
High school, no diploma	37 (10.3%)	6 (16.2%)		16 (43.2%)	
High school diploma	19 (5.3%)	3 (15.8%)		6 (31.6%)	
Some college, no diploma	13 (3.6%)	1 (7.7%)		2 (15.4%)	
Some university, no diploma	148 (41.1%)	25 (16.9%)		55 (37.2%)	
College diploma	14 (3.9%)	4 (28.6%)		6 (42.9%)	
Bachelor’s diploma and higher	109 (30.3%)	16 (14.7%)		46 (42.2%)	
Employment status			0.500		0.078
Employed full-time	31 (8.6%)	7 (22.6%)		19 (61.3%)	
Employed part-time	27 (7.5%)	2 (7.4%)		10 (37.0%)	
Self-employed	21 (5.8%)	4 (19.0%)		6 (28.6%)	
Unemployed	17 (4.7%)	1 (5.9%)		4 (23.5%)	
Student	248 (68.9%)	40 (16.1%)		90 (36.3%)	
Unable to work	2 (0.6%)	0 (0.0%)		1 (50.0%)	
Marital status			0.196		0.888
Married/Living with a partner	24 (6.7%)	7 (29.2%)		9 (37.5%)	
Single^(b)^	273 (75.8%)	41 (15.0%)		104 (38.1%)	
Other	41 (11.4%)	7 (17.1%)		14 (34.1%)	
Immigration status			0.213		0.248
Citizen	46 (12.8%)	10 (21.7%)		23 (50.0%)	
LPR^(c)^	64 (17.8%)	5 (7.8%)		20 (31.3%)	
CPR^(d)^	13 (3.6%)	2 (15.4%)		5 (38.5%)	
Non-immigrant^(e)^	213 (59.2%)	36 (16.9%)		79 (37.1%)	
Linguistic preference	1.73 ± 0.70	1.81 ± 0.83	0.407	1.76 ± 0.73	0.448
Social preference	1.53 ± 0.74	1.68 ± 0.85	0.139	1.58 ± 0.78	0.338
Perceived discrimination	4.89 ± 4.00	7.02 ± 4.74	<0.001	5.16 ± 3.97	0.322
Compassion	5.35 ± 1.81	5.82 ± 1.69	0.032	5.88 ± 1.68	<0.001
Total	360 (100%)	57 (15.8%)		134 (37.2%)	

*(a) Other countries included were: Argentina, Australia, Canada, Finland, France, Germany, Italy, Korea, Malaysia, Mongolia, Netherlands, New Zealand, Philippines, Russia, Singapore, Sweden, Thailand, United Arab Emirates, and Vietnam; (b) Single (Never married /Widowed /Divorced /Separated); (c) LPR: Legal Permanent Resident (“green card holder,” or “blue card holder”); (d) CPR: Conditional Permanent Resident; and (e) Non-immigrant (e.g., visitors for business and for pleasure, students, temporary workers and trainees, treaty traders and investors, exchange visitors, religious workers, etc.).*

### Volunteering and Charitable Giving

57 (15.8%) and 134 (37.2%) of participants engaged in volunteering and charitable giving during the pandemic, respectively. (Table 1).

Using Chi-square tests and Student *t*-tests, we detected participation rate in volunteering was different by country of residence (*P* = 0.003) and policy stringency (*P* = 0.006), while only country of residence (*P* = 0.013) influenced charitable giving. Participation in volunteering ranged from 24.7% in Chinese youths residing in countries including Argentina, Australia, Canada, Finland, France, Germany, Italy, Korea, Malaysia, Mongolia, Netherlands, New Zealand, Philippines, Russia, Singapore, Sweden, Thailand, United Arab Emirates, and Vietnam, followed by 21.2% in the United States, Japan (15.6%), the United Kingdom (10.3%), and Spain (5.8%). Policy stringency indexes were significantly lower in the residential countries of volunteers (71.76 ± 14.01) compared to those who were not (77.15 ± 13.29). 51.9% of youths in Argentina, Australia, Canada, Finland, France, Germany, Italy, Korea, Malaysia, Mongolia, Netherlands, New Zealand, Philippines, Russia, Singapore, Sweden, Thailand, United Arab Emirates, and Vietnam overall gave charitable donations for COVID relief, followed by those in the United States (39.4%), Japan (31.3%), Spain (28.8%), and the United Kingdom (28.2%).

### Association Between Compassion, Perceived Discrimination, and Prosocial Behaviors

**FIGURE 1:**
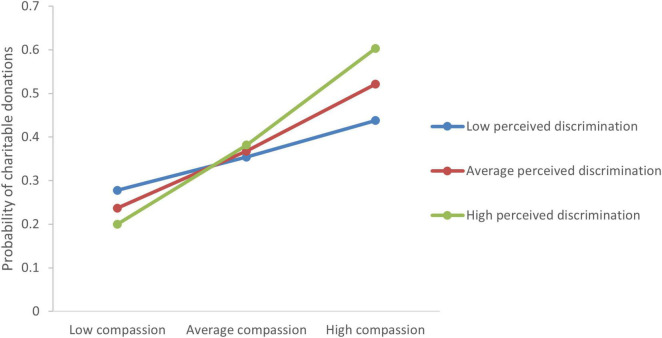
Perceived discrimination enhances the positive effect of compassion on charitable giving.

When controlling for all sociodemographic covariates, the analysis showed positive main effects of compassion (*B* = 0.22, *P* = 0.037) and perceived discrimination (*B* = 0.16, *P* < 0.001) on volunteering (Model fit: Nagelkerke R^2^ = 0.233). There was no interaction between perceived discrimination and compassion on youths’ volunteering (*B* = 0.02, *P* = 0.388, Nagelkerke R^2^ = 0.195). Only compassion (*B* = 0.34, *P* < 0.001) but not perceived discrimination (*B* = 0.01, *P* = 0.696) positively associated with charitable giving (Nagelkerke R^2^ = 0.183), while perceived discrimination showed a marginal trend for enhancing the effect of compassion on participation in charitable giving (*B* = 0.04, *P* = 0.062, Nagelkerke R^2^ = 0.150). (Figure 1). Notably, employment status affected probability of participation in charitable giving, with self-employed (*B* = −1.42, *P* = 0.047) and unemployed persons (*B* = −1.56, *P* = 0.037) and students (*B* = −1.05, *P* = 0.028) participating less in donations than those who were employed full-time.

## Discussion

This study found compassion as prosocial behaviors’ proximal predictor in adolescents and young adults. The role of perceived discrimination is also salient in youths’ prosociality, as it independently predicted participation in volunteering, and could potentially enhance the association between compassion and charitable giving, which partly confirmed our first and second hypotheses. This adds to the evidence that prosociality in ethnic minority youths are sensitive to social context, and that racial discrimination is an indispensable part in migrant and immigrant youths’ prosocial development.

Consistent with accumulating research on the association between compassion and helping in adolescents and young adults, this study shows compassion-related helping in diasporic Chinese youths is prevalent during the COVID-19 pandemic. A study done in 2004–2005, prior to the pandemic, found annual volunteer rate in Chinese American was 18.8% (Sundeen et al., 2007), which is significantly lower than that in our study (21.2%), which included a much shorter time span of less than 5 months and only for COVID-19 relief purposes. Additionally, it is notable that no socioeconomic variables, except employment status, affected participation in charitable giving, and their effect sizes (self-employed, β = 0.24; unemployed, β = 0.21; student, β = 0.35) are minimal compared with compassion (β = 1.41). This adds to the evidence that compassion could be a strong motivation among young people for helping others, and indicates that the pandemic might present as an opportunity for cultivating and extending global compassion (Peplak and Malti, 2021).

This study is the first to examine the effect of discrimination on prosocial behaviors in youths of Chinese descent. McGinley et al. (2009) found that acculturative stress is positively related with various prosocial tendencies in Mexican American college students. Brittian et al. (2013) and Davis et al. (2021) found discrimination as a longitudinal predictor of public prosocial behaviors among Mexican American adolescents. In addition, discrimination’s effects differ regarding types of helping behaviors, indicating these two specific forms of helping (volunteering and charitable giving) may be qualitatively different. One possible explanation is that volunteering is considered more “public,” which is often exhibited in the presence of others and is related directly to image and reputation (Exley, 2018), while donating is usually done anonymously without the knowledge of others (McGinley et al., 2009). Kraus and Callaghan’s research confirmed that public and private contexts moderated prosocial tendencies as public scenarios tend to make reputational concerns more salient (Kraus and Callaghan, 2016). It is possible that Chinese youths engaged in charitable activities to combat stereotypes related to their racial identity inflamed by the COVID-19 pandemic. It is also possible that prosocial engagement transpires from resilience of racial discrimination.

However, it should be noted that although perceived discrimination is positively related to adolescents and young adults’ prosocial engagement in this study, according to Smart Richman and Leary (2009), when people perceive that there is a high probability of restoring relationship, prosocial responses should occur, but if this assessment were to change, then more antisocial responses would be expected. Thus, elimination of persisting racial discrimination, restoration of relationships, as well as acknowledgment and recognition of their prosocial efforts in pandemic control, should be given to diasporic Chinese in order to extend their prosociality and lay the foundation for their future participation in civil society.

This study has several limitations. First, due to limited time to capture timely data as well as the globally distributed nature of the population under investigation, the sample was not entirely randomized, raising concern of sample coverage and volunteer bias. For example, participants under 18 years old were underrepresented, probably because of their relatively limited access to online questionnaires. However, we minimized the sociodemographic variations in prosocial participation in analyses controlling for confounding factors. Second, the questionnaire implemented did not ask the respondents to describe the details of their experience of engagement in prosocial behaviors. This study would be strengthened by qualitative, follow-up studies that sought to elucidate the reasons underlying studied factors. Lastly, the cross-sectional design of this study could not provide us with causal relations, for example, bidirectional relationships between prosocial behavior and compassion has been recognized (Carlo et al., 2015). This could be attained by future longitudinal studies.

## Conclusion

Diasporic Chinese living in countries other than China faced open discrimination during the pandemic. Chinese youths’ prosocial engagement for COVID-19 relief was influenced by perceived discrimination as well as compassion. Understanding and extending prosociality among this population has implications for fighting immediate and future crisis, and building a more integrated community. This study provides insights for understanding ethnic minority youths’ prosociality by considering how compassion interacts with social contexts such as racial discrimination to shape prosocial decisions and potentially influence prosocial development.

## Data Availability Statement

The raw data supporting the conclusions of this article will be made available by the authors, without undue reservation.

## Ethics Statement

The studies involving human participants were reviewed and approved by the Ethics Committee of Wenzhou Medical University. Written informed consent from the participants’ legal guardian/next of kin was not required to participate in this study in accordance with the national legislation and the institutional requirements.

## Author Contributions

YC: conceptualization, data curation, formal analysis, investigation, methodology, resources, software, and writing—original draft. ZW: conceptualization, investigation, resources, and writing—original draft. QZ and WD: conceptualization, resources, and writing—review and editing. JX and SW: investigation and resources. XZ: resources, investigation, and writing—review and editing. CC: conceptualization, funding acquisition, resources, supervision, and writing—review and editing. All authors contributed to the article and approved the submitted version.

## Conflict of Interest

The authors declare that the research was conducted in the absence of any commercial or financial relationships that could be construed as a potential conflict of interest.

## Publisher’s Note

All claims expressed in this article are solely those of the authors and do not necessarily represent those of their affiliated organizations, or those of the publisher, the editors and the reviewers. Any product that may be evaluated in this article, or claim that may be made by its manufacturer, is not guaranteed or endorsed by the publisher.
