# Supplementary material for: Compassion, Discrimination, and Prosocial Behaviors: Young Diasporic Chinese During the COVID-19 Pandemic
Source: Front Psychol. 2022 Feb 17;13:814869. doi: 10.3389/fpsyg.2022.814869 (PMC8891152; doi:10.3389/fpsyg.2022.814869)
Supplement: Supplementary file 1 [file Table_1.DOCX]

Supplementary Table 1 Multiple Regression Results of Perceived Discrimination and Compassion on Volunteering and Charitable giving

| **Predictors** | **Volunteering** | | **Charitable giving** | |  |
| --- | --- | --- | --- | --- | --- |
|  | B (SE) | β | B (SE) | β |  |
| **Gender** |  |  |  |  |  |
| Male | REF | NA | REF | NA |  |
| Female | 0.02 (0.37) | 1.02 | -0.21 (0.27) | 0.81 |  |
| **Age** |  |  |  |  |  |
| < 18 | REF | NA | REF | NA |  |
| 18-25 | -0.57 (0.7) | 0.57 | -0.48 (0.56) | 0.62 |  |
| **Country of residence** |  |  |  |  |  |
| The United States | REF | NA | REF | NA |  |
| Spain | -1.36 (0.84) | 0.26 | -0.44 (0.6) | 0.64 |  |
| The United Kingdom | -0.04 (0.72) | 0.96 | -0.48 (0.52) | 0.62 |  |
| Japan | 0.34 (0.73) | 1.41 | 0.2 (0.59) | 1.23 |  |
| Others ^(b)^ | 0.66 (0.47) | 1.93 | 0.6 (0.39) | 1.82 |  |
| **Stringency** | -0.02 (0.02) | 0.99 | 0.02 (0.02) | 1.02 |  |
| **Educational level** | -0.09 (0.13) | 0.91 | 0.08 (0.09) | 1.08 |  |
| **Employment status** |  |  |  |  |  |
| Employed full-time | REF | NA | REF | NA |  |
| Employed part-time | -0.5 (0.97) | 0.61 | -0.46 (0.61) | 0.63 |  |
| Self-employed | 0.55 (0.88) | 1.73 | -1.42 (0.72) | 0.24* |  |
| Unemployed | -0.72 (1.2) | 0.49 | -1.56 (0.75) | 0.21* |  |
| Student | 0.25 (0.66) | 1.28 | -1.05 (0.48) | 0.35* |  |
| Retired | -18.97 (28398.88) | 0 | - | - |  |
| Unable to work | - | - | -0.52 (1.58) | 0.6 |  |
| **Marital status** |  |  |  |  |  |
| Married/ Living with a partner/ Common law | REF | NA | REF | NA |  |
| Single ^(a)^ | -0.51 (0.68) | 0.6 | 0.91 (0.58) | 2.47 |  |
| Other | -0.77 (0.96) | 0.46 | 0.96 (0.7) | 2.6 |  |
| **Immigration status** |  |  |  |  |  |
| Citizen | REF | NA | REF | NA |  |
| LPR ^(c)^ | -0.46 (0.75) | 0.63 | -1.06 (0.48) | 0.35 |  |
| CPR ^(d)^ | -0.42 (1.02) | 0.66 | -0.26 (0.73) | 0.77 |  |
| Non-immigrant ^(e)^ | -0.83 (0.55) | 0.44 | -0.78 (0.46) | 0.46 |  |
| **Linguistic preference** | 0.19 (0.26) | 1.21 | 0.14 (0.21) | 1.15 |  |
| **Social preference** | 0.41 (0.26) | 1.51 | 0.16 (0.2) | 1.17 |  |
| **Perceived discrimination** | 0.16 (0.05) | 1.17*** | 0.01 (0.03) | 1.01 |  |
| **Compassion** | 0.22 (0.11) | 1.25* | 0.34 (0.08) | 1.41*** |  |
| **Model-fit index** | Nagelkerke R^2^ = 0.233 | | Nagelkerke R^2^ = 0.183 | |  |

*p *<* 0.05. **p *<* 0.01. ***p < 0.001.

(a) Other countries included were: Argentina, Australia, Canada, Finland, France, Germany, Italy, Korea, Malaysia, Mongolia, Netherlands, New Zealand, Philippines, Russia, Singapore, Sweden, Thailand, United Arab Emirates, and Vietnam; (b) Single (Never married /Widowed /Divorced /Separated); (c) LPR: Legal Permanent Resident (“green card holder,” or “blue card holder”); (d) CPR: Conditional Permanent Resident; (e) Non-immigrant (e.g., visitors for business and for pleasure, students, temporary workers and trainees, treaty traders and investors, exchange visitors, religious workers, etc.)
